# Supplementary material for: An open-source framework for large-scale, flexible evaluation of biomedical text mining systems
Source: J Biomed Discov Collab. 2008 Jan 29;3:1. doi: 10.1186/1747-5333-3-1 (PMC2276192; doi:10.1186/1747-5333-3-1)
Supplement: Additional file 1 — Evaluation platform source code. The additional file contains the software evaluation framework discussed in this paper. The most current release can be downloaded from SourceForge.net [1]. See the accompanying README file for instructions on installation and use. Also included in the distribution is a collection of UIMA wrappers for some commonly used BioNLP tools and annotated corpora. [file 1747-5333-3-1-S1.zip › bionlp_uima_repository-1.0.1/docs/api/edu/uchsc/ccp/util/nlp/mention/comparison/IdenticalClassMentionComparator.html]

IdenticalClassMentionComparator (Center for Computational Pharmacology Util/UIMA API)


|  |  |  |
| --- | --- | --- |
| **Overview** \| **Package** \| **Class** \| **Tree** \| **Deprecated** \| **Index** \| **Help** | |  |
| **PREV CLASS**   **NEXT CLASS** | **FRAMES**    **NO FRAMES**     **All Classes** |  |
| SUMMARY: NESTED \| FIELD \| CONSTR \| METHOD | DETAIL: FIELD \| CONSTR \| METHOD |  |


---


## edu.uchsc.ccp.util.nlp.mention.comparison Class IdenticalClassMentionComparator

```
java.lang.Object
  edu.uchsc.ccp.util.nlp.mention.comparison.MentionComparator
      edu.uchsc.ccp.util.nlp.mention.comparison.IdenticalMentionComparator
          edu.uchsc.ccp.util.nlp.mention.comparison.IdenticalClassMentionComparator
```

**All Implemented Interfaces:**: ClassMentionComparator, Comparator<Mention>

---

``` public class IdenticalClassMentionComparator extends IdenticalMentionComparator implements ClassMentionComparator ```

This class implements a comparator which compares ClassMentions. ClassMentions must be identical.

**Author:**
:   williamb

---

**Field Summary**

**Fields inherited from class edu.uchsc.ccp.util.nlp.mention.comparison.MentionComparator**

`MULTIPLIER`


**Constructor Summary**

`IdenticalClassMentionComparator()`


| **Method Summary** | |
| --- | --- |
| `int` | `compare(ClassMention cm1, ClassMention cm2, SpanComparator spanComparator, int maximumComparisonDepth)` |
| `int` | `compare(Mention mention1, Mention mention2, SpanComparator spanComparator, int maximumComparisonDepth, int depth)`             Compare two mentions. |

**Methods inherited from class edu.uchsc.ccp.util.nlp.mention.comparison.IdenticalMentionComparator**

`hasEquivalentMentionNames`

**Methods inherited from class edu.uchsc.ccp.util.nlp.mention.comparison.MentionComparator**

`compare, equalComplexSlotMentions, equalSlotMentions`

**Methods inherited from class java.lang.Object**

`clone, equals, finalize, getClass, hashCode, notify, notifyAll, toString, wait, wait, wait`

**Methods inherited from interface java.util.Comparator**

`equals`

**Constructor Detail**

### IdenticalClassMentionComparator

```
public IdenticalClassMentionComparator()
```


**Method Detail**

### compare

```
public int compare(Mention mention1,
                   Mention mention2,
                   SpanComparator spanComparator,
                   int maximumComparisonDepth,
                   int depth)
```

:   Compare two mentions. They must be ClassMentions or else an UnsupportedOperationException is thrown.

    :   **Specified by:**: `compare` in class `MentionComparator`

---


### compare

```
public int compare(ClassMention cm1,
                   ClassMention cm2,
                   SpanComparator spanComparator,
                   int maximumComparisonDepth)
```

:   **Specified by:**: `compare` in interface `ClassMentionComparator`
:   **Parameters:**: `cm1` -: `cm2` -: `spanComparator` -: `maximumComparisonDepth` - **Returns:**


---


|  |  |  |
| --- | --- | --- |
| **Overview** \| **Package** \| **Class** \| **Tree** \| **Deprecated** \| **Index** \| **Help** | |  |
| **PREV CLASS**   **NEXT CLASS** | **FRAMES**    **NO FRAMES**     **All Classes** |  |
| SUMMARY: NESTED \| FIELD \| CONSTR \| METHOD | DETAIL: FIELD \| CONSTR \| METHOD |  |


---
